# Network-based differential gene expression analysis suggests cell cycle related genes regulated by E2F1 underlie the molecular difference between smoker and non-smoker lung adenocarcinoma

**DOI:** 10.1186/1471-2105-14-365

**Published:** 2013-12-17

**Authors:** Chao Wu, Jun Zhu, Xuegong Zhang

**Affiliations:** 1 MOE Key Laboratory of Bioinformatics and Bioinformatics Division, TNLIST and Department of Automation, Tsinghua University, Beijing 100084, PR China; 2 Department of Genetics and Genomic Sciences, Icahn Institute of Genomics and Multiscale Biology, Mount Sinai School of Medicine, New York, NY, USA

## Abstract

**Background:**

Differential gene expression (DGE) analysis is commonly used to reveal the deregulated molecular mechanisms of complex diseases. However, traditional DGE analysis (e.g., the t test or the rank sum test) tests each gene independently without considering interactions between them. Top-ranked differentially regulated genes prioritized by the analysis may not directly relate to the coherent molecular changes underlying complex diseases. Joint analyses of co-expression and DGE have been applied to reveal the deregulated molecular modules underlying complex diseases. Most of these methods consist of separate steps: first to identify gene-gene relationships under the studied phenotype then to integrate them with gene expression changes for prioritizing signature genes, or vice versa. It is warrant a method that can simultaneously consider gene-gene co-expression strength and corresponding expression level changes so that both types of information can be leveraged optimally.

**Results:**

In this paper, we develop a gene module based method for differential gene expression analysis, named network-based differential gene expression (nDGE) analysis, a one-step integrative process for prioritizing deregulated genes and grouping them into gene modules. We demonstrate that nDGE outperforms existing methods in prioritizing deregulated genes and discovering deregulated gene modules using simulated data sets. When tested on a series of smoker and non-smoker lung adenocarcinoma data sets, we show that top differentially regulated genes identified by the rank sum test in different sets are not consistent while top ranked genes defined by nDGE in different data sets significantly overlap. nDGE results suggest that a differentially regulated gene module, which is enriched for cell cycle related genes and E2F1 targeted genes, plays a role in the molecular differences between smoker and non-smoker lung adenocarcinoma.

**Conclusions:**

In this paper, we develop nDGE to prioritize deregulated genes and group them into gene modules by simultaneously considering gene expression level changes and gene-gene co-regulations. When applied to both simulated and empirical data, nDGE outperforms the traditional DGE method. More specifically, when applied to smoker and non-smoker lung cancer sets, nDGE results illustrate the molecular differences between smoker and non-smoker lung cancer.

## Background

High throughput technologies enable people to monitor the transcriptome of complex diseases. It’s a great opportunity as well as a big challenge for us to reveal the deregulated molecular mechanisms of complex diseases from transcriptomic data. Over the decade, differential gene expression (DGE) analysis has been widely used to discover differentially regulated genes and deregulated molecular mechanisms [1]. However, changes in multiple genes coupled with interactions among themselves and between them and other genes interfere normal biological functions of cell and cause diseases [2]. Traditional DGE analysis such as the t test or the rank sum test doesn’t always perform well on identifying deregulated genes and deregulated molecular mechanisms because it processes each gene independently without considering gene-gene relationships [3,4]. Genes most significantly differentially regulated might not directly relate to diseases. More suitable tools need to be developed for identifying the deregulated molecular mechanisms from transcriptomic data.

Functional genomics studies reveal that genes and their products are well governed in cell: they are elaborately assembled and disassembled by regulatory forces beyond genetic code [5]. Revealing gene-gene relationships among differentially regulated genes and identifying causal relationships or gene modules in them will lead to a better understanding of deregulated molecular mechanisms and discovery of potential causal factors. Many efforts have been devoted to integrate gene-gene relationships and gene expression level changes to prioritize signature genes, such as some gene prioritization methods and gene module based methods [3,6-13]. However, most of the methods involve multiple separate steps in defining gene expression changes and gene-gene relationships related to disease without maximally leveraging all information available simultaneously.

In this work, we extend our previously developed Networked Gene Prioritizer (NGP) method [10] and develop a gene module based method for differential gene expression analysis, named network-based differential gene expression (nDGE) analysis to prioritize deregulated genes and group them into gene modules. NGP leverages a protein-protein interaction network and differential expressed genes in a network neighborhood to prioritize genes. Improvement of nDGE comparing to NGP and other existing methods is that it uses a one-step integrative process to simultaneously define gene-gene relationships and gene expression level changes associated with diseases while most existing methods involve two separated steps to define them. The resulted advantage is that no hard cutoff parameters are needed in nDGE to determine neither gene-gene relationships nor gene expression changes associated with disease while hard cutoff parameters are needed in most existing methods and might lead the methods sensitive to the selection of parameters. No hard cutoff parameters are needed in NGP, either. However, NGP’s ability to prioritize all the genes on chips is limited because it relies on protein-protein interaction network which only covers a fraction of whole proteome. In addition, NGP might not be able to accurately prioritize some genes because of limitations of its underlie assumption that a physical interaction implies a co-expression relationship.

We first compare nDGE with a traditional DGE, NGP and two gene module based methods using simulated data sets. The DGE is carried out by the rank sum test. One version of gene module based methods in our comparison is to construct co-expression network using only differentially expressed genes and detecting modules within it. The other version is to apply co-expression analysis using all genes in data sets, then to extract co-expression gene modules and apply gene set enrichment analysis to identify the differentially expressed gene modules. We demonstrate that nDGE outperforms the compared methods on accuracy of prioritizing deregulated genes and identifying deregulated gene modules with a large range of parameter of co-expression measurement.

Then, we apply nDGE to a series of smoker and non-smoker lung adenocarcinoma data sets to explore the molecular mechanisms and regulators that drive differences between smoker and non-smoker lung adenocarcinoma. Lung cancer is the most common cancer in terms of both incidence and mortality. In 2008, there were 1.61 million new cases, and 1.38 million deaths due to lung cancer [14]. Tobacco smoke is the most common cause of lung cancer. But non-smokers account for 10–15% of lung cancer cases [15]. This percentage is even higher in Asian women [16]. Although studies have suggested that lung cancers arising in non-smokers have a distinct natural history, profile of oncogenic mutations, and response to targeted therapy comparing to smokers [17], lung cancer in smokers and non-smokers is treated similarly to date. Identifying the molecular mechanisms and capturing the regulatory factors that explain the differences between smoker and non-smoker lung cancer can extend our understanding of smoking and non-smoking related lung cancer and will provide benefits for the treatment of lung cancer. We apply nDGE and the rank sum test on multiple smoker and non-smoker lung cancer data sets. Top differentially regulated genes identified by the rank sum test in different sets are not consistent while top ranked genes defined by nDGE in different data sets significantly overlap. A differentially regulated gene module identified by nDGE, which is enriched for cell cycle genes and E2F1 targeted genes, plays a role attributing to the differences between smoker and non-smoker lung adenocarcinoma. Existing data support that E2F1 regulates cell cycle genes that lead to the molecular differences associated with different response to chemotherapies between smoker and non-smoker lung adenocarcinoma. In conclusion, our nDGE results provide a better understanding of smoker and non-smoker lung cancers which can lead to better early lung cancer detection and personalized treatment of smoker and non-smoker lung cancer.

## Methods

### Data sets

Six types of simulated data sets and six smoker and non-smoker lung adenocarcinoma data sets are used in this paper. Information about the smoker and non-smoker lung adenocarcinoma data sets is listed in Table 1. For Smoker1 and Smoker5 data sets, probe sets whose intensities are less than log2(40) in more than 20% samples are filtered. 18981 probe sets representing 11563 Entrez genes in Smoker1 data set and 32761 probe sets representing 14904 Entrez genes in Smoker5 data set are used for further analysis. For Smoker3 and Smoker6 data sets, probes whose detected *p* values are larger than 0.05 in at least 20% of samples are filtered, and 14230 probes representing 10908 Entrez genes in Smoker3 data set and 14297 probes for 11293 Entrez genes in Smoker6 data set are retained for further analysis; in Smoker2 and Smoker4 data sets which are based on two-color microarray, all probes are used for further analysis.

**Table 1:** Information about the empirical data used in this work

**Data set**	**GEO ID**	**Sample subtypes**	**Platform**	**Normalization**
Smoker1	gse10072	42(smoker):16(non-smoker)	Affy133a	Quantile
Smoker2	gse11969	44(smoker):45(non-smoker)	Agilent Homo sapiens 21.6 K custom array	Loess
Smoker3	gse29016	28(smoker):9(non-smoker)	Illumina HumanHT-12 V3.0	Quantile
Smoker4	gse26939	69(smoker):7(non-smoker)	Agilent-UNC-custom-4X44K	Loess
Smoker5	gse31210	111(smoker):115(non-smoker)	Affy133plus2	Quantile
Smoker6	gse32863	29(smoker):29(non-smoker)	Illumina HumanWG-6 v3.0	Quantile

**Figure 1:**
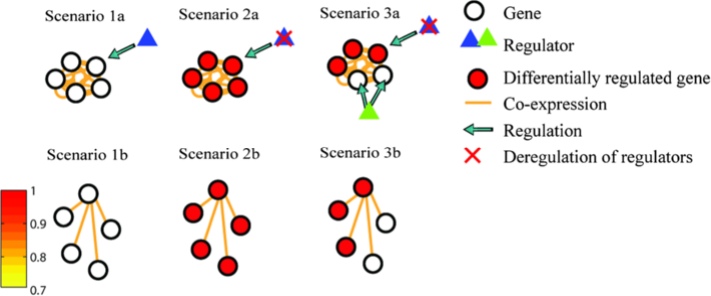
**A toy model of potential regulatory programs in complex diseases.** The figure illustrates the potential regulatory programs in complex diseases-Scenario 1a, 2a and 3a. A gene in each regulatory program is randomly selected. Differential expression pattern of the co-expressed neighbors of the genes is illustrated in Scenario 1b, 2b and 3b respectively. The colour bar indicates co-expression strength between the co-expressed genes.

Six types of data sets are generated in simulation experiments to simulate the scenarios that might occur in the cellular regulatory programs which might directly relate to diseases. The six types of data sets are called data sets 1–6 for the convenience of description. For each type of data set, we generate 100 simulation examples. We compare nDGE with NGP using simulated data sets 1 and 2. In data set 1, a gene with differentially expressed co-regulated neighbors that are common in two different subtypes and subtype-specific is created. Some of the neighbors are co-expressed with the candidate gene in both subtypes of samples and some are co-expressed with the candidate gene in only one subtype of samples. In data set 2, a gene whose co-regulated neighbors are differentially expressed is created. We compare nDGE with the rank sum test using data set 3 where a differentially expressed co-expressed gene module is simulated. Using simulated data sets 4–6, we compare nDGE with two co-expression based methods. In data set 4, we simulate a scenario that the regulator of a gene module is not dysregulated between subtypes of disease (Scenario 1a in Figure 1). As a result, genes in the gene module are not differentially expressed so that their co-regulated neighbors are not differentially expressed, either (Scenario 1b in Figure 1). In data set 5, dysregulation of a regulator leads to the differential expression of its target genes (Scenario 2a in Figure 1). Thus, genes in the gene module are differentially expressed as well as their co-regulated neighbors (Scenario 2b in Figure 1). In data set 6, we simulate a complex scenario where the regulator of a gene module is dysregulated but only part of its target genes are differentially expressed (Scenario 3a in Figure 1). As a result, a gene with differentially expressed neighbors and non-differentially expressed neighbors is generated (Scenario 3b in Figure 1). This is in line with the redundancy principle in gene regulation which indicates that genes might be regulated by multiple other regulators to keep their expression stable when their master regulator is deregulated [18]. More details about the simulated data sets are given in Additional file 1.

### Methodology of nDGE

**Figure 2:**
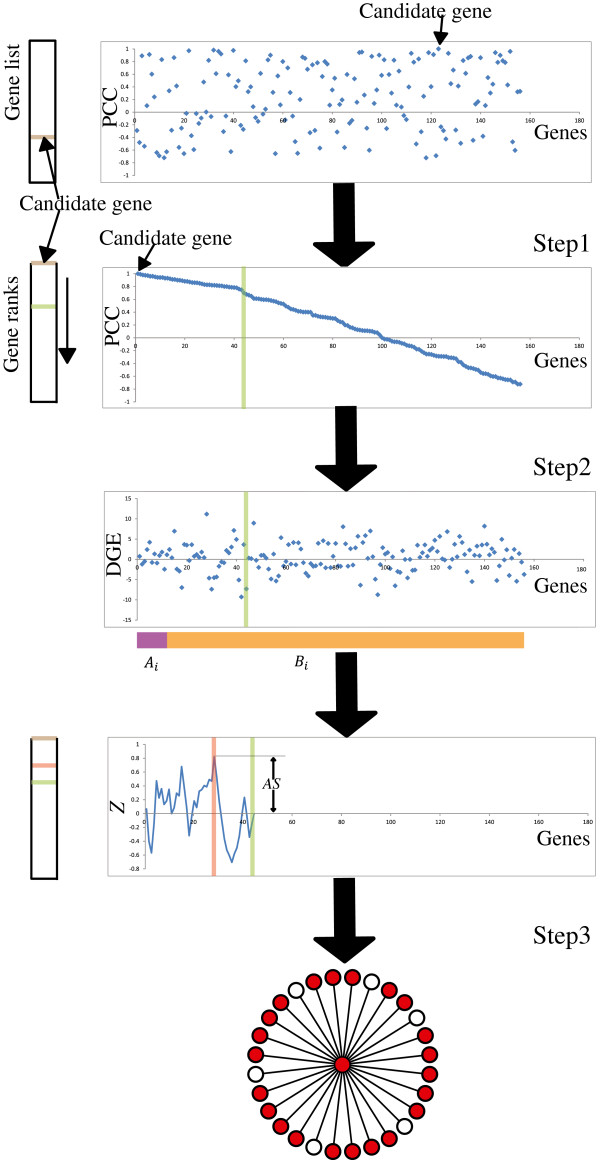
**Flowchart of nDGE.** In step 1, we identify neighbors of the candidate probe set. In step 2, we calculate activity score (*AS*) of the candidate probe set. In step 3, we identify DE neighbors of the candidate probe set.

nDGE contains 3 steps (flowchart showed in Figure 2). All pairwise Pearson Correlation Coefficients (*PCCs*) of probe sets’ in each subtype of samples are calculated. Although both positive and negative expression correlations between genes are employed to infer gene-gene relationships in literatures, we focus on positively correlated (co-expressed) genes as co-regulated neighbors in nDGE.

Step 1- For each candidate probe set, all probe sets on the chips are sorted by their *PCC*s with the candidate probe set. We identify its co-regulated probe sets by their values of *PCC*. We assume that the probe sets are co-regulated if they are highly co-expressed in a subtype of samples (at *PCC* > 0.707).

Step 2 - We calculate probe sets’ differential expression between the compared samples as *Z* score of the rank sum test statistic assuming the rank sum test statistic is normally distributed. We calculate activity score (*AS*) of candidate probe set as following: at first, we walk in the co-regulated probe sets (the probe sets before the green line in Figure 2) step by step; in each step we generate two gene sets-*A*_*i*_ and *B*_*i*_ (the colored bar in Figure 2), *A*_*i*_ contains the top-*i* probe sets, *B*_*i*_ contains the other probe sets; next, we test whether the differential expression of genes in *A*_*i*_ and *B*_*i*_ is different by the rank sum test. *AS* of the candidate probe set is formulated as the product of the *Z* score of rank sum test statistic and a correction factor:

$$\mathrm{AS}={\left(-1\right)}^{\alpha }x\;\text{ma}x_{i\in \text{neighbor}}\left(\frac{W_{i}-u_{i}}{\sigma_{i}}x\frac{W_{i}-u_{i}}{W_{\max}-u_{i}}\right)$$,

where $W_{i}=\sum_{j\in A_{i}}\text{rank}\left(z_{j}\right)$, $u_{i}=\frac{i\left(N+1\right)}{2}$, $\sigma_{i}=\sqrt{\frac{i\left(N-i\right)\left(N+1\right)}{12}}$, *Z*_*j*_ is the *Z* score of probe sets’ differential expression by the rank sum test, *N* is the number of probe sets on the chip, $\frac{W_{i}-u_{i}}{\sigma_{i}}$ is the *Z* score of the rank sum test statistic which shows whether the differential expression of genes in *A*_*i*_ and *B*_*i*_ is different, $\frac{W_{i}-u_{i}}{W_{\max}-u_{i}}$ is the ratio of the true *Z* score of the candidate gene with the largest possible *Z* of the candidate gene, which is used as a correction factor of the *Z* score for adjusting different set sizes of co-regulated neighbors. If $\frac{W_{i}-u_{i}}{\sigma_{i}}>0$ parameter *a* = 0, else *a* = 1.

Step 3 – For each probe set, their co-regulated neighbors that contribute to *AS* are defined as its differentially expressed neighbors (DE neighbors).

Statistical significance of *AS* is estimated by permutation tests. We keep the size of co-regulated neighbors of a candidate probe set the same but randomly select its neighbors from probes on the chip. If the true *AS* of a candidate probe set is negative, then we calculate the “negative *AS*” which is the minimum of ${\left(-1\right)}^{\alpha }\;x\;\frac{W_{i}-u_{i}}{\sigma_{i}}x\frac{W_{i}-u_{i}}{W_{\max}-u_{i}}$, where $W_{i}=\sum_{j\in A_{i}}\text{rank}\left(z_{j}\right)$, *A*_*i*_ is the aforementioned neighbors set, *Z*_*j*_ is the *Z* score of probe sets’ differential expression by the rank sum test, $u_{i}=\frac{i\left(N+1\right)}{2}$, $\sigma_{i}=\sqrt{\frac{i\left(N-i\right)\left(N+1\right)}{12}}$, $W_{\text{max}}=\sum_{j=0}^{i-1}\;N-j$, *N* is the number of probe sets on the chip, if $\frac{W_{i}-u_{i}}{\sigma_{i}}>0$ parameter *a* = 0, else *a* =1.

If *AS* of a candidate probe set is positive, then we calculate the “positive *AS*” which is the maximum of ${\left(-1\right)}^{\alpha }x\;\frac{W_{i}-u_{i}}{\sigma_{i}}x\frac{W_{i}-u_{i}}{W_{\text{max}}-u_{i}}$.

We repeat the aforementioned process 100,000 times to generate negative (or positive) *AS* background distributions. At last, *p* value is estimated as the frequency of *AS* background smaller (or larger) than the true *AS*.

Two prioritization lists are returned in nDGE, one is for treatment samples and the other is for control samples. Different co-expression and differential gene expression patterns might exist in the different types of samples which suggest different regulatory programs might be deregulated in the different subtypes of disease. For this reason, we independently prioritize genes in treatment and control samples and draw conclusions in each subtype of samples.

More details about nDGE are discussed in Additional file 1. Please refer Additional file 1 for the detail.

### Identification of differentially regulated gene modules

nDGE prioritizes deregulated genes and groups them into gene modules. At first, deregulated genes are extracted according to their *p* value; then, DE neighbor relationships between these genes are extracted; next, interactions indicating genes are DE neighbors of each other are retained to construct a gene-gene network; at last, if the network is densely connected we employ a spectrum clustering algorithm [19] followed by a coherence-based module detection algorithm [20] to identify gene modules, else we define gene modules as connected components in sparse network.

### Comparison of nDGE with two gene module based methods

A straightforward approach to identify differentially regulated gene modules is to determine co-expression gene modules first and then to inspect differential expression of genes in the modules or vice versa. We name the approach as two-steps approach. A problem of two-steps approach is that gene modules are determined by the parameter of co-expression measurement without leveraging the information of differential gene expression. The selection of the parameter for co-expression modules will affect the differentially regulated gene module results. Here we develop nDGE which simultaneously considers gene-gene co-expression and differential gene expression to identify differentially regulated gene modules. Thus, the variation of co-expression parameter has few impacts on the results.

We compare nDGE with two two-steps methods. One approach, noted as method 1, is to construct co-expression network of differentially expressed genes and detecting modules within it. An alternative approach is first to apply co-expression analysis on all the genes to identify co-expression gene modules then to apply GSEA [13] to identify modules enriched for differentially expressed genes. We apply the leading edge analysis (LEA) to GSEA result to further refine the identified gene modules. We note the method as method 2.

We compare nDGE with method 1 and 2 on simulated data sets 4–6. Performances of the methods on revealing deregulated gene modules are measured by whether they can identify the candidate genes and their differentially expressed neighbors in the simulated data sets. In each data set, the number of true co-regulated neighbors is 50. The “detected” co-regulated neighbors are set as 50, 100, 150, 200, 250, 300, 350, 400, 450 and 500. In data set 4, any neighbors identified by the methods are regarded as false positives. In data sets 5 and 6, the overlapped genes and unique genes of the detected neighbors by the methods with the truly differentially expressed neighbors are counted and their ratio is defined as a true positive rate.

No hard cutoff parameter is needed in nDGE and method 2 to determine differential gene expression while a cutoff parameter is needed in method 1. In order to fairly compare the three methods on the variation of co-expression parameter, we set top 1–100 genes as the differentially expressed genes in method 1, which is close to the true differentially expressed neighbors in data sets 5 and 6. In method 2, the *p* parameter of GSEA is set 0 and co-regulated neighbors are identified by LEA analysis of GSEA.

### Function and regulator analyses for differentially regulated gene modules

Functional annotation analysis is carried out to investigate the potential biological functions of modules identified by nDGE. The tool we used is “Functional Annotation Clustering” analysis of DAVID [21], the parameter is default except enrichment thresholds is set as 0.0001 and Bonferroni corrected *p* < 0.01. Only Gene Ontology terms are showed in the results. Regulator analysis is implemented to discover potential regulators of modules. The tool we used is “Transcription Factor Target Analysis” of WebGestalt (WEB-based GEne SeT AnaLysis Toolkit) [22] and the parameter is set as Bonferroni corrected *p* < 0.01.

## Result

We first compare nDGE with traditional DGE which is carried out by the rank sum test, NGP and two gene module based methods using simulated data sets. nDGE outperforms NGP on accurately prioritizing deregulated genes (see Additional file 1 for the detail). Here we focus on comparing nDGE with other existing methods. Then we apply nDGE to a series of smoker and non-smoker lung adenocarcinoma data sets to reveal the molecular differences between smoker and non-smoker lung cancer.

### nDGE outperforms the traditional DGE on gene prioritization

Differential gene expression (DGE) analysis is widely used to reveal the deregulated molecular mechanisms underlying complex diseases from transcriptomic data. However, traditional DGE analysis (e.g., the t test or the rank sum test) tests each gene independently without considering interactions between them. Developing a method that performs better than currently available DGE methods is one of motivations of nDGE. nDGE and the rank sum test are applied on simulated data set 3. *Z* score of the rank sum test statistic is taken as the measurement of differential gene expression. The Spearman Correlation Coefficient (*SCC*) between *Z* score and *AS* of candidate genes without co-expressed neighbors is calculated. The *SCC* is always 1 in 100 simulation experiments. Thus, nDGE returns the same prioritization list as the rank sum test when candidate genes have no co-expressed neighbors. The ranks of candidate genes that have differentially expressed co-regulated neighbors are counted. The 50 candidate genes that are co-expressed and differentially expressed are always ranked at 1^th^ to 50^th^ in the prioritization list in 100 simulation experiments. The candidate genes whose neighbors are differentially expressed are top-ranked in nDGE. Comparing to the rank sum test analysis, nDGE sets a higher rank for genes that are co-expressed and differentially expressed than genes that are only differentially expressed. We think this feature enables nDGE to more accurately capture coherently deregulated genes because co-expression relationships among genes reflect co-regulation relationships among them [12,23]. Top-prioritized genes by nDGE are likely to involve in deregulated regulatory programs of the studied disease.

### nDGE outperforms two gene module based methods on discovering deregulated gene modules

Differentially regulated gene modules in transcriptome level may shed light on the dysregulated molecular mechanisms underlying complex diseases. A straightforward approach to identify these gene modules is to determine co-expression gene modules first and then to inspect differential expression of genes in the modules or vice versa. For example, in GSEA software [13], a set of co-expression gene modules centered on cancer-related genes have been defined in MigSDB and used to test whether they are differentially expressed in query data sets. In WGCNA software [11], gene significance measurement is based on biological significance of the identified co-expression gene modules such as the correlation with clinical traits. The problem of two-steps approach is that the information of gene-gene co-expression and differential gene expression is not maximally leveraged. Thus the variation of the parameter of co-expression measurement will influence the differentially regulated gene module results. We compare nDGE with two simple and straightforward two-steps methods which are named methods 1 and 2 (see “Methods” for the details).

**Figure 3:**
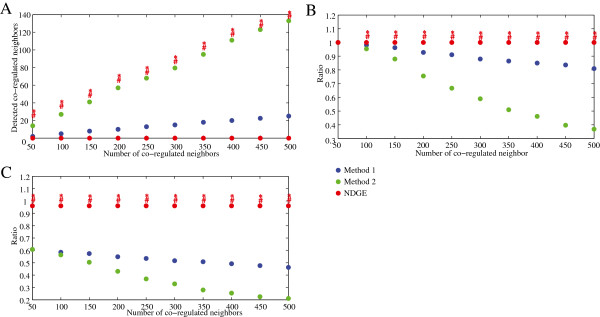
**Performances of nDGE and methods 1 and 2 in simulated data sets. A,** the median of false positive genes detected by the methods in data set 4 in 100 simulation experiments. **B,** the median of the ratio of true positive genes revealed by the methods in data set 5 in 100 simulation experiments. **C,** the median of the ratio of true positive genes discovered by the methods in data set 6 in 100 simulation experiments. *indicates the genes (or the ratios of genes) detected by nDGE are significantly larger/smaller than that revealed by method 1 with *p* < 0.05 (paired t test). #indicates the genes (or the ratios of genes) detected by nDGE are significantly larger/smaller than that revealed by method 2 with *p* < 0.05 (paired t test).

We apply nDGE and method 1 and 2 in simulated data sets 4–6 with a range of co-expression parameters. Numbers of co-regulated neighbors in the simulation sets are 50, 100, 150, 200, 250, 300, 350, 400, 450 and 500. No co-regulated genes are expected in data set 4. nDGE detects less false positive genes than method 1 and 2 with different sizes of co-regulated neighbors in 100 simulation experiments (Figure 3A). Fifty differentially expressed co-regulated neighbors of the candidate gene are expected in data 5. nDGE reveals the most true-positive genes among tested methods in 100 simulation experiments (Figure 3B). In data set 6, we consider the redundancy principle in gene regulation that genes might be regulated by other regulators to keep their expression stable when their master regulator is deregulated [18]. Only 30 of 50 co-regulated neighbors of a candidate gene are differentially expressed. nDGE reveals the most true neighbor genes among test methods in 100 simulation experiments (Figure 3C).

In all tests, nDGE has high sensitivity in revealing deregulated co-regulated genes and high specificity without detecting many false positives across a large range of co-expression parameters. Altogether, simulation results suggest that nDGE robustly performs better than currently available methods in term of detecting coherently differentially regulated genes.

### Molecular differences between smoker and non-smoker lung adenocarcinoma

Lung cancers arising in non-smokers and smokers are different diseases. But they are treated similarly to date. Discovering the molecular mechanisms that lead to the differences between smoker and non-smoker lung cancer will extend our understanding of lung cancer and provide benefits for risk evaluation for early lung cancer detection and personalized treatment of different lung cancers. nDGE is applied to multiple smoker and non-smoker lung adenocarcinoma data sets (listed in Table 1). It is applied to non-smoker samples in Smoker2, Smoker5 and Smoker6 data sets because sizes of non-smoker samples in the other data sets are small, limiting the power of nDGE to infer reliable co-expression relationships. We make notation conventions on some conceptual designations: probe sets whose DE neighbors are of higher expression levels in smoker samples are regarded as “upregulated”; probe sets whose DE neighbors have lower expression levels in smoker samples are noted as “downregulated”. We assign genes’ *AS* by *AS* of their probe sets that have the largest absolute *AS*. The rank sum test is also applied in the data sets and its prioritization results are taken as reference for comparison. *Z* score of the rank sum test statistic is taken as the statistic. Genes with higher expression levels in smoker samples are regarded as upregulated genes; the ones with lower expression levels in smoker samples are downregulated genes. Genes’ differential expression level is assigned by *Z* score of their probe sets whom have the largest absolute *Z* score.

#### Top-ranked genes by the rank sum test and nDGE are different

**Figure 4:**
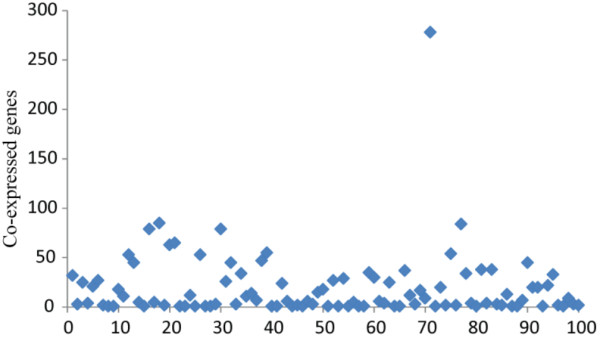
**Co-expressed genes of top-ranked genes by the rank sum test.** The co-expressed genes are determined by *PCC* > 0.707.

Overlap ratios between two top 50 (also 100 and 200) gene sets prioritized by the rank sum test and nDGE in smoker samples are shown in Table 2. The result indicates that top-prioritized genes by the rank sum test and nDGE are different. To further illustrate the differences, we investigate co-expression patterns of top 100 upregulated genes identified by the rank sum test in smoker samples of Smoker1 data set. It is shown that the top-ranked genes have few co-expressed genes (Figure 4). Most of them are independent from each other.

**Table 2:** Overlap ratios of the top-ranked genes by the rank sum test and nDGE

**Data set**	**Up-regulated genes**			**Down-regulated genes**		
	Top50*	Top100	Top200	Top50	Top100	Top200
Smoker1	0.2	0.19	0.185	0	0	0.065
Smoker2	0	0.01	0.04	0	0	0.035
Smoker3	0	0	0.035	0	0.03	0.025
Smoker4	0	0.02	0.145	0.02	0.03	0.075
Smoker5	0	0.04	0.14	0	0.08	0.16
Smoker6	0.02	0.06	0.1	0	0.01	0.05

*overlap between the top-50 genes prioritized by DGE and nDGE are counted as *A*, and then overlap ratio is calculated as *A/50*.

#### Consistency of top ranked gene sets derived from different data sets

**Figure 5:**
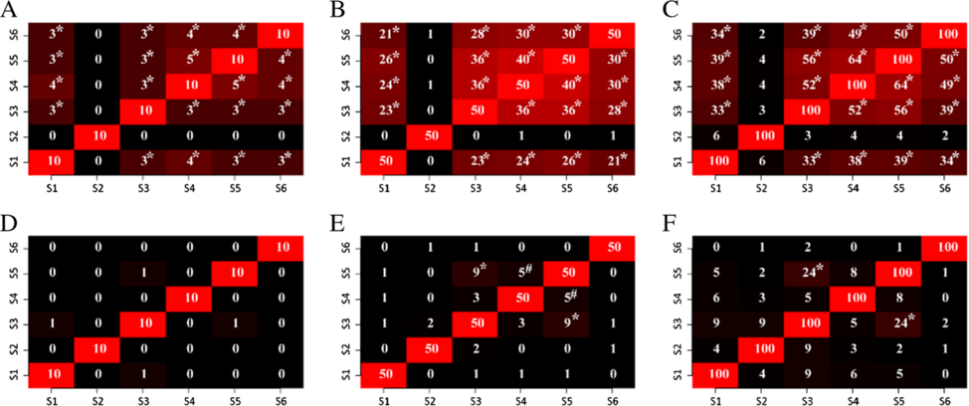
**Overlaps between two top deregulated gene lists by nDGE in smoker samples of the different data sets.** The S1 to S6 represent the data sets Smoker1 to Smoker6. “#” and “*” indicate the number of overlap is significantly larger than that in permutation background with *P* < 0.00005 and *P* < 0.00001, respectively. **A** to **C** show overlaps of top 10, 50 and 100 up regulated genes between the different data sets. **D** to **F** show overlaps of top 10, 50 and 100 downregulated genes between the different data sets.

**Figure 6:**
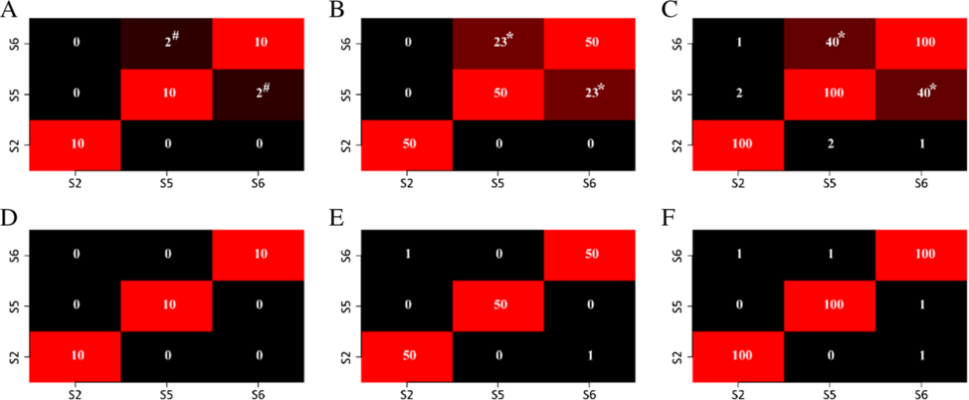
**Overlaps between two top deregulated gene lists by nDGE in non-smoker samples of the different data sets.** The S2, S5 and S6 represent the data sets Smoker2, Smoker5 and Smoker6. “#” and “*” indicate the number of overlap is significantly larger than that in permutation background with *P* < 0.00005 and *P* < 0.00001, respectively. **A** to **C** show overlaps of top 10, 50 and 100 up regulated genes between the different data sets. **D** to **F** show overlaps of top 10, 50 and 100 downregulated genes between the different data sets.

**Figure 7:**
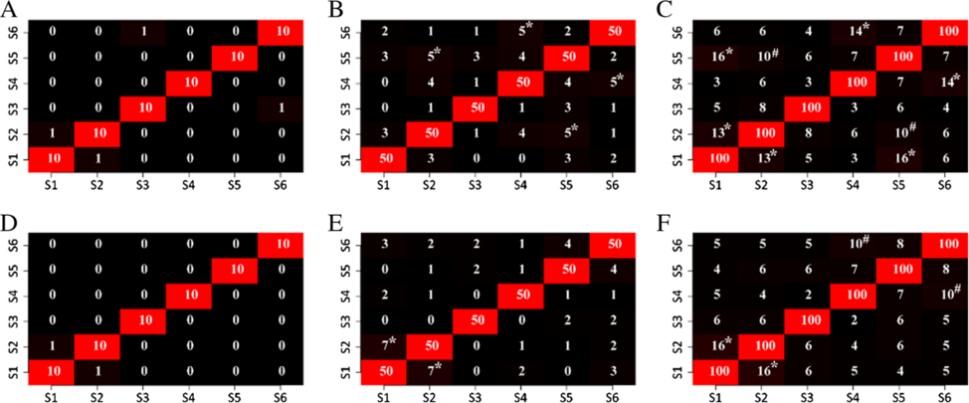
**Overlaps between two top deregulated gene lists by the rank sum test.** The S1 to S6 represent the data set Smoker1 to Smoker6. “#” and “*” indicate the number of overlap is significantly larger than that in permutation background with *P* < 0.00005 and *P* < 0.00001, respectively. **A** to **C** show overlaps of top 10, 50 and 100 up regulated genes between different data sets. **D** to **F** show overlaps of top 10, 50 and 100 downregulated genes between different data sets.

Top upregulated genes prioritized in different data sets by nDGE are highly consistent while top-ranked genes identified by the rank sum test in different data sets do not significantly overlap. Five thousand and thirty-two genes are spotted in all 6 microarray data sets. Overlaps between top 10 (also 50 and 100) genes prioritized by nDGE in smoker samples in the different data sets are counted (Figure 5). The top upregulated genes in smoker samples of Smoker1, Smoker3, Smoker4, Smoker5 and Smoker6 data sets are consistent, but the top downregulated genes in the data sets are different. Six thousand three hundred and eighty-two genes are spotted in Smoker2, Smoker5 and Smoker6 microarray data sets. Overlaps between top 10 (also 50 and 100) genes prioritized by nDGE in non-smoker samples in the data sets are counted (Figure 6). Similarly, the top upregulated genes between Smoker5 and Smoker6 data sets are consistent while the top downregulated genes in the data sets are different. Overlaps between top 10 (also 50 and 100) genes prioritized by the rank sum test in the different data sets are counted (Figure 7). Neither the top upregulated genes nor the top downregulated genes are consistent.

#### Integrative analysis

**Figure 8:**
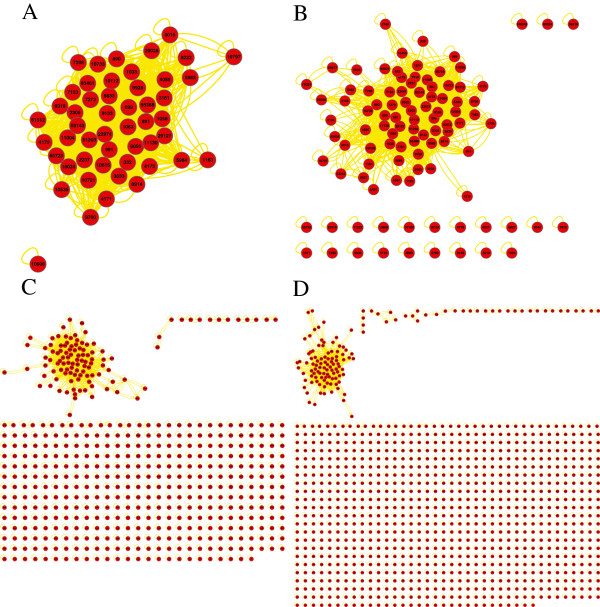
**Consensus networks of the top upregulated genes in smoker samples.** The consensus networks of top 50 genes **(A)**, top 100 genes **(B)**, top 500 genes **(C)** and top 1,000 genes **(D)** are shown.

We further apply integrative analysis on the top upregulated genes prioritized by nDGE in smoker samples of Smoker1, Smoker3, Smoker4, Smoker5 and Smoker6 data sets as they are consistent to each other. Five thousand and thirty-two genes are ordered by their average ranks in the data sets. Then top 50 (also 100, 500 and 1,000) upregulated genes are extracted. Gene networks of these top ranked gene sets are constructed respectively. Interactions that exist in more than two data sets are used to construct consensus networks. The consensus networks are visualized in Cytoscape [24] using yFile organic layout algorithm. We identify gene modules as connected components in these sparse networks. There is one module in the networks (Figure 8) which suggests that the module might play a role in smoker samples contributing to the molecular differences between smoker and non-smoker samples.

We select the module in the top 100 gene network (Additional file 2: Table S1) as the representative module to apply function and regulator analyses because: 1) most of genes in the network are in the module; and 2) the module consists of core components of modules in the other networks (Table 3). Cell cycle related genes as well as E2F1 targeted genes are enriched in the module (Additional file 3: Table S2 and Additional file 4: Table S3).

**Table 3:** Percentages of modules occupying their networks and the other modules in smoker samples

**Gene modules#**	**Genes in the module**	% of the network*	**% of the top 50 gene module**	**% of the top 100 gene module**	**% of the top 500 gene module**	**% of the top 1000 gene module**
Top 50	49	98%	100%	63.6%	49.5%	48.5%
Top 100	77	77%	100%	100%	77.8%	76.2%
Top 500	99	19.8%	100%	100%	100%	98.2%
Top 1000	101	10.1%	100%	100%	100%	100%

#The “Top 50” etc. is the short name for the gene module in the top 50 gene network.

*This column indicates the percentage that the modules occupy their networks.

**Figure 9:**
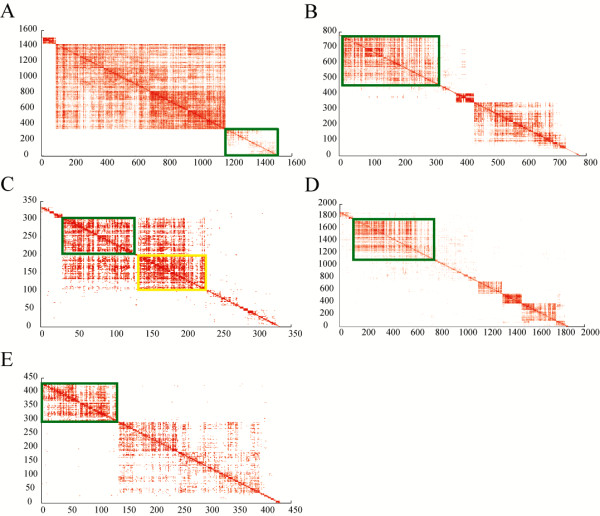
**Upregulated gene module that enriches for cell cycle related genes and E2F1 target genes exists in smoker samples in each data set.** The modules that enrich for cell cycle related genes and E2F1 target genes in smoker samples of Smoker1 dataset **(A)**, Smoker3 dataset **(B)**, Smoker4 dataset **(C)**, Smoker5 dataset **(D)** and Smoker6 dataset **(E)** are highlighted in the figures.

Upregulated gene modules are also detected in the five data sets respectively to check whether the module discovered by integrative analysis exists in each data set or not. The significant upregulated genes (or probe sets, probes) (*p* < 0.000001) in each data set are extracted. Upregulated gene modules in the densely connected networks are identified using the spectral clustering [19] followed by the coherence-based module identification procedure [20]. The gene module enriched for cell cycle related genes and E2F1 targeted genes is consistently identified in every data set (Figure 9, Additional file 5: Table S4 and Additional file 6: Table S5). Two gene modules enriched for cell cycle related genes and E2F1 target genes are identified in Smoker4 data set. However, there are dense inter-module interactions suggesting that the two modules function as one coherent gene module. The analyses together suggest that E2F1 up-regulates the cell cycle related gene module in smoker samples.

**Figure 10:**
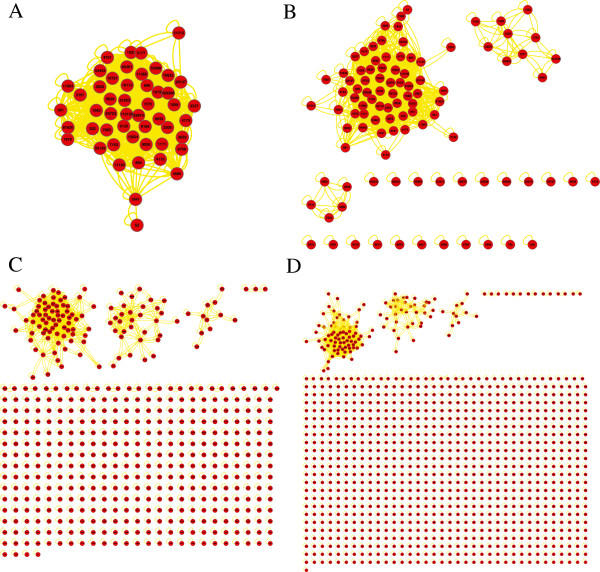
**Consensus networks of the top upregulated genes by nDGE in non-smoker samples. A,** consensus network of top 50 genes. **B,** consensus network of top 100 genes. **C,** consensus network of top 500 genes. **D,** consensus network of top 1,000 genes.

nDGE’s result is subtype-specific. It is worth to also investigate deregulated gene modules in non-smoker samples. Integrative analysis is applied to the upregulated genes prioritized by nDGE in non-smoker samples of Smoker5 and Smoker6 data sets. Six thousand three hundred and eighty-two genes are ordered by their average ranks in the data sets. Then top 50 (also 100, 500 and 1,000) upregulated genes are extracted. Gene networks of these top ranked gene sets are constructed respectively. Interactions that are discovered in both data sets are used to construct consensus networks. The consensus networks are visualized in Cytoscape using yFile organic layout algorithm. There is a module common in the four networks (the module in Figure 10A and the largest module in Figure 10B, 10C and 10D) suggesting that it plays a role contributing to the molecular differences between smoker and non-smoker lung adenocarcinoma.

**Figure 11:**
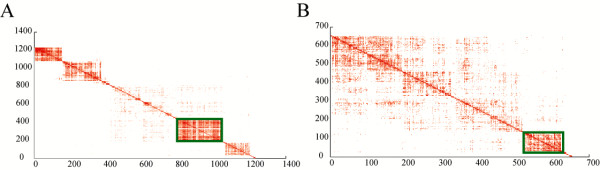
**Upregulated gene module that enriches for cell cycle related genes and E2F1 targeted genes in non-smoker samples of Smoker5 and Smoker6 data sets.** The modules that enrich for cell cycle related genes and E2F1 targeted genes in non-smoker samples of Smoker5 dataset **(A)** and Smoker6 dataset **(B)** are highlighted in the figures.

Again, we select the module in the top 100 gene network (Additional file 7: Table S6) as the representative module because: 1) most of genes in the network are in the module; and 2) the module consists of the major component of modules in the other networks (Table 4). It is revealed that cell cycle related genes and E2F1 targeted genes are enriched in the module (Additional file 8: Table S7 and Additional file 9: Table S8). The analysis on each data set confirms the results of integrative analysis (Figure 11, Additional file 10: Table S9 and Additional file 11: Table S10).

**Table 4:** Percentages of modules occupying their networks and the other modules in non-smoker samples

**Gene modules#**	**Genes in the module**	% in the network*	**% in the top 50 gene module**	**% in the top 100 gene module**	**% in the top 500 gene module**	**% in the top 1000 gene module**
Top 50	50	100%	100%	79.4%	66.7%	65.8%
Top 100	63	63%	100%	100%	84%	82.9%
Top 500	75	15%	100%	100%	100%	98.7%
Top 1000	76	7.6%	100%	100%	100%	100

#The “Top 50” etc. is the short name for the gene module in the top genes networks.

*This column indicates the percentages that the genes occupy their networks.

The analyses suggest that E2F1 might regulate cell cycle related genes in smoker and non-smoker samples and play a role contributing to the molecular differences between smoker and non-smoker lung adenocarcinoma. Forty-seven genes are in the overlap between the 77-gene module identified in the top 100 gene network in smoker samples and the 63-gene module identified in the top 100 gene network in non-smoker samples. E2F1 might partially explain the molecular differences between smoker and non-smoker samples by up-regulating the genes in smoker samples and down-regulating them in non-smoker samples.

#### A potential molecular mechanism

**Figure 12:**
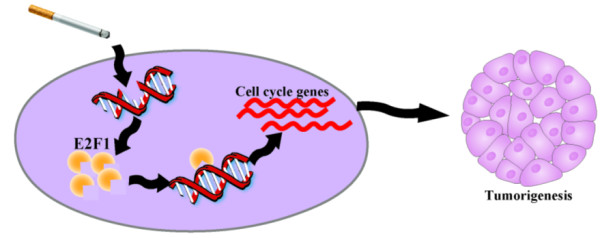
A possible mechanism of smoker lung adenocarcinoma.

E2F1 plays a crucial role in controlling cell cycle and interacts with tumor suppressor proteins; it binds preferentially to retinoblastoma protein pRB in a cell-cycle dependent manner; and it mediates both cell proliferation and p53-dependent/independent apoptosis. However, the role it plays in cancer is paradox: it can be an oncogene or a tumor suppressor [25]. Recent studies reveal that E2F1 undergoes posttranslational modifications in response to DNA damage, resulting in E2F1 stabilization [26]. The accumulated E2F1 protein promotes cell cycle progression, particularly G1/S transition and contributes to tumorigenesis [27]. DNA adducts and DNA damage caused by carcinogen in cigarette is regarded as one of main mechanisms that lead to cancer caused by smoking [28]. These literatures suggest that smoking leads to DNA damage, and then DNA damage causes the accumulation of E2F1, which in turn activates cell cycle progression and contributes to tumorigenesis in smokers. This process is one of the factors that cause lung adenocarcinoma in smoker and partially explain the molecular differences between smoker and non-smoker lung adenocarcinoma (Figure 12).

It is shown that lung cancer patients who have never smoked respond better to chemotherapy than smoker lung cancer patients [29]. Down regulation of E2F1 enhances the sensitivity of chemotherapy of cancer cell [30-32]. Our nDGE result demonstrates that the gene module regulated by E2F1 is up-regulated in smoker samples and down-regulated in non-smoker samples. E2F1 might have higher activity in smoker samples than in non-smoker samples. Altogether, these results suggest that the gene module regulated by E2F1 might explain the different response to chemotherapies between smoker and non-smoker lung cancers.

## Discussion

Lung cancer is the most common cause of cancer-related death in men and women worldwide [14]. Although it has been suggested that lung cancer arising in non-smokers and smokers have distinct natural history, profile of oncogenic mutations, and response to targeted therapy [17], lung cancer in smokers and non-smokers is treated similarly to date. In our work, the integrative analysis of a series of smoker and non-smoker lung adenocarcinoma data sets shows that E2F1 might regulate a gene module enriched for cell cycle related genes, in turn, partially explain the molecular differences between smoker and non-smoker lung adenocarcinoma and different response to chemotherapies between smoker and non-smoker lung adenocarcinoma. The result leads to a better understanding of smoking and non-smoking related lung cancer and may provide benefits for risk evaluation for early lung cancer detection and personalized treatment of different lung cancers.

In this work, we develop a gene module based differential gene expression analysis, named network-based differential gene expression (nDGE) to prioritize differentially regulated genes and group them into gene modules. The key improvement of nDGE comparing to currently available methods is that nDGE uses a one-step integrative process to simultaneously identify gene-gene relationships and gene expression level changes associated with diseases while most existing methods involve two separated steps to define them. The resulted advantage is that no hard cutoff parameters are required in nDGE to determine gene-gene relationships and gene expression level changes associated with disease.

DGE analysis has been widely used in transcriptomic analysis of complex diseases. However, the traditional DGE analysis such as the rank sum test or the t test doesn’t always perform well in discovering differentially regulated genes and deregulated molecular mechanisms because the most significantly differentially expressed genes may not directly associate with the deregulated regulatory programs of the studied disease. The rank sum test analysis on the smoker and non-smoker lung adenocarcinoma data sets explicates it. The coherently deregulated genes together might better reflect the deregulated regulatory programs of complex disease. nDGE is developed aiming to identify these coherently deregulated genes.

## Conclusions

In this work, we develop nDGE to prioritize deregulated genes and group them into gene modules. When applied to both simulated and empirical data, nDGE outperforms currently available methods in term of detecting coherently deregulated genes. More specifically, when applied to smoker and non-smoker lung cancer sets, nDGE results elucidate the molecular differences between smoker and non-smoker lung cancers that lead to different response to chemotherapies. We hope the result will lead to a better understanding of smoking and non-smoking related lung cancer and provide benefits for risk evaluation for early lung cancer detection and personalized treatment of different lung cancers.

## Competing interests

The authors declare that they have no competing interests.

## Authors’ contributions

CW collected the data, designed and conducted the study, and drafted the manuscript. JZ led the study, participated in the design of the study, and drafted the manuscript. XZ led the study, participated in the design of the study and help to draft the manuscript. All authors read and approved the final manuscript.

## Supplementary Material

Additional file 1 The supplemental materials that describe the simulated data sets, the development of nDGE and the advantages of nDGE comparing to NGP. Click here for file

Additional file 2: Table S1 Genes in the module of the top 100 gene network in smoker samples. Click here for file

Additional file 3: Table S2 Functional annotation of the module in the top 100 gene network in smoker samples. Click here for file

Additional file 4: Table S3 Motif analysis of the module in the top 100 gene network in smoker samples. Click here for file

Additional file 5: Table S4 Function annotation of the module enriched for cell cycle related genes in smoker samples of each data set. Click here for file

Additional file 6: Table S5 Motif analysis of the modules that is enriched for cell cycle related genes in smoker samples of each data set. Click here for file

Additional file 7: Table S6 Genes in the module of the top 100 genes network in non-smoker samples. Click here for file

Additional file 8: Table S7 Function annotation of the module in the top 100 gene network in nonsmoker samples. Click here for file

Additional file 9: Table S8 Motif analysis of the module in the top 100 gene network in nonsmoker samples. Click here for file

Additional file 10: Table S9 Function annotation of the module enriched for cell cycle related genes in non-smoker samples of each data set. Click here for file

Additional file 11: Table S10 Motif analysis of the modules that are enriched for cell cycle related genes in non-smoker samples of Smoker5 and Smoker6 data sets. Click here for file
