# Supplementary material for: Network-based differential gene expression analysis suggests cell cycle related genes regulated by E2F1 underlie the molecular difference between smoker and non-smoker lung adenocarcinoma
Source: BMC Bioinformatics. 2013 Dec 17;14:365. doi: 10.1186/1471-2105-14-365 (PMC3878503; doi:10.1186/1471-2105-14-365)
Supplement: Additional file 1 — The supplemental materials that describe the simulated data sets, the development of nDGE and the advantages of nDGE comparing to NGP. [file 1471-2105-14-365-S1.doc]

# Supplementary Materials for

# Network-based differential gene expression analysis suggests cell cycle related genes regulated by E2F1 underlie the molecular difference between smoker and non-smoker lung adenocarcinoma

### Chao Wu^1^, Jun Zhu^2*^, Xuegong Zhang^1^

^1^MOE Key Laboratory of Bioinformatics and Bioinformatics Division, TNLIST and Department of Automation, Tsinghua University, Beijing 100084, PR China

^2^Department of Genetics and Genomic Sciences, Icahn Institute of Genomics and Multiscale Biology, Icahn Medical School at Mount Sinai, New York, New York, United States of America

^*^Corresponding author

Email addresses:

CW: [wuchao1984@gmail.com](mailto:wuchao1984@gmail.com)

JZ: [jun.zhu@mssm.edu](mailto:jun.zhu@mssm.edu)

XZ: [zhangxg@tsinghua.edu.cn](mailto:zhangxg@tsinghua.edu.cn)

# Generation of simulated data sets

Six types of data sets are generated in simulation experiments. The six types of data sets are called data sets 1-6 for the convenience of description. For each type of data set, we generate 100 simulation examples. The simulated data sets 1 and 2 are used for comparing network-based differential gene expression (nDGE) analysis with networked gene prioritizer (NGP). The simulated data set 3 is used for comparing nDGE with the rank sum test. The simulated data sets 4-6 are used for comparing nDGE with two gene module based methods. All simulations are based on the Smoker5 data set because it has the largest sample size. In our simulations, we randomly selected 2,000 probe sets for each simulation, which can represent a mixture of complex regulations. Two thousand probe sets in Smoker5 data set are randomly selected. Their expression levels between smoker and non-smoker samples are preserved, but their co-expression relationships between them in each subtype are destroyed by permuting expression levels of each gene. These probes are sorted by the rank sum test statistics. In simulated data set 1, we select the top 100^th^ differentially expressed probe set, then simulate its co-expressed neighbors in smoker samples using the probe sets ranked between 101 and 200 and its co-expressed neighbors in non-smoker samples using the probe sets ranked between 101 and 150. Thus 50 neighbors of the probe set are common between two subtypes and its other 50 neighbors are subtype-specific. In simulated data set 2, we simulate co-expressed neighbors of the top 100^th^ probe set using the probe sets ranked between 101 and 150 in smoker samples. Spearman Correlation Coefficients (*SCCs*) of the regulator probe set with other probe sets are randomly distributed with regard to probe sets’ differential expression measurement. In simulated data set 3, we simulate a co-expression module in smoker samples consisting of the top 50^th^ to 99^th^ differentially expressed probe sets. In simulated data set 4, we select the top 100^th^ differentially expressed probe set and simulate its co-expressed neighbors with other 50 probe sets in smoker samples. Differential expression measurement of the probe sets in the data set is randomly distributed with regard to their *SCCs* with the selected gene. In simulated data set 5, we select the top 100^th^ differentially expressed probe set and simulate its co-expressed neighbors with the probe sets ranked between 50 and 99 in smoker samples. Differential expression measurement of other probe sets is randomly distributed with regard to their *SCCs* with the selected gene. In simulated data set 6, we select the top 100^th^ differentially expressed probe set and simulate its co-expressed neighbors with the probe sets ranked between 50 and 79 and between 801 and 820. Differential expression measurement of other probe sets in the data set is randomly distributed with regard to their *SCCs* with the probe set ranked at top 100^th^. One hundred simulation examples are generated for each type of simulated data set.

# Development of nDGE

We discuss two questions about nDGE here: 1) why co-expressed genes but not expression correlated genes are selected as co-regulated neighbors; 2) how activity score (*AS*) is proposed?

## Why co-expressed genes but not expression correlated genes are selected as co-regulated neighbors?

Both positively and negatively correlated genes are included in inferring gene-gene relationships in many published papers. Why only positively correlated (co-expressed) genes are selected as co-regulated neighbors in nDGE?

nDGE prioritizes genes by their neighbors’ differential expression. Candidate genes are scored by the proposed *AS*. The goal of the method is to select the neighbor set giving candidate genes higher *AS* when more than one neighbor set is available.

We test nDGE with two different neighbor sets in the smoker and non-smoker lung adenocarcinoma data sets: set *A* is co-expressed genes and set *B* is expression correlated genes. However we need to make some modifications on nDGE before applying it with set *B*. In step 1 of nDGE, genes are ordered by their absolute Pearson correlation coefficients (*PCCs*) and co-regulated neighbors are selected by absolute *PCC*>0.707; in step 2, genes’ differential expression is measured by absolute *Z* scores of the rank sum test statistic which means upregulated and downregulated genes are not distinguished. In nDGE with set *A*, positive *AS* indicates that candidate genes’ DE neighbors are upregulated, negative *AS* shows that they are downregulated. In nDGE with set *B*, positive *AS* indicates that candidate genes’ DE neighbors are deregulated (either upregulated or downregulated), negative *AS* shows that they are not deregulated. Our goal is to select the set giving candidate genes higher positive *AS* in the smoker and non-smoker lung adenocarcinoma data sets.

The results show that co-expressed genes are better than expression correlated genes because most candidate genes get larger positive *AS* in set *A* than in set *B* (Supplementary Figure 1). Thus we select co-expressed genes as co-regulated genes.


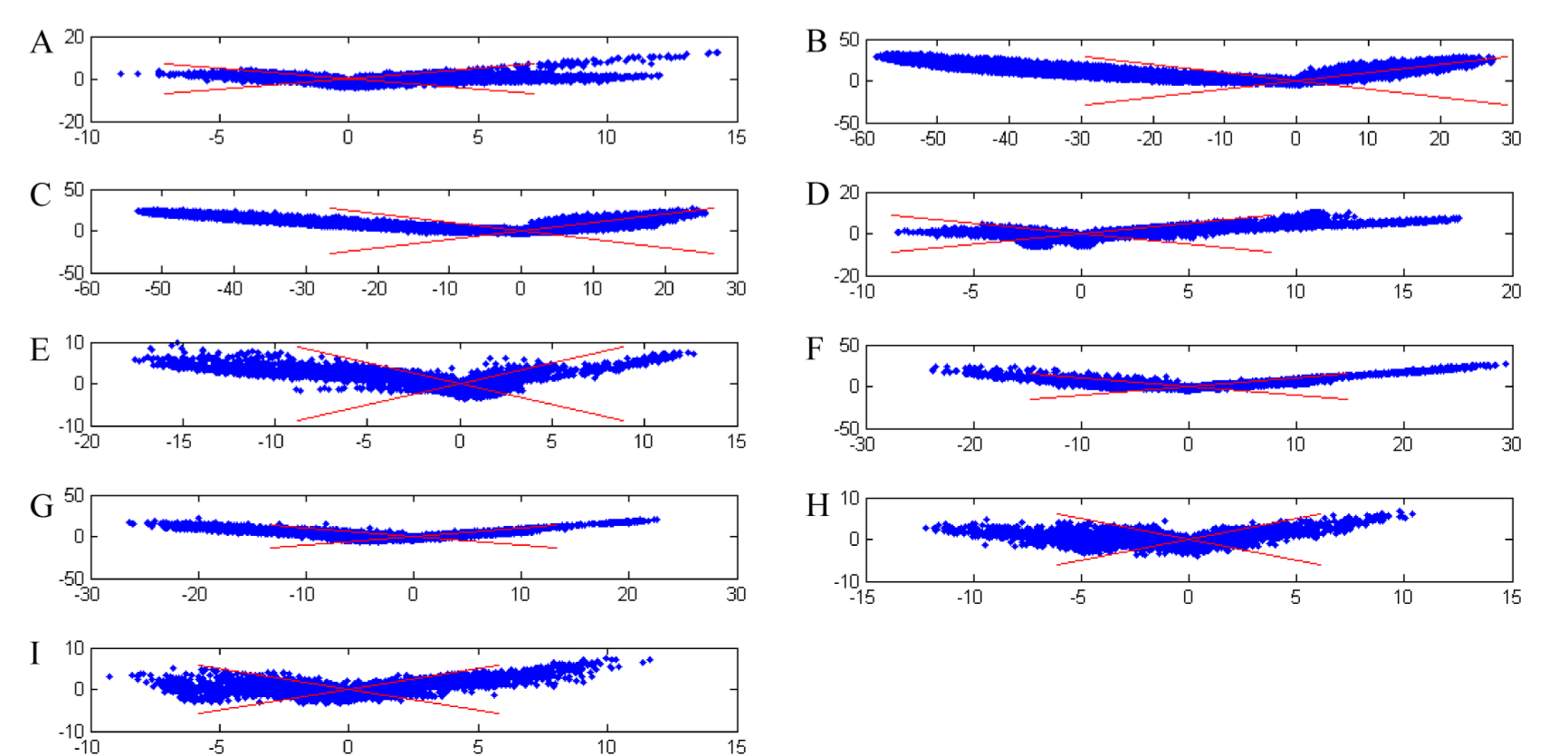


**SFigure 1 *AS* of genes with different gene sets as neighbors**

X axis shows *AS* of candidate genes with set *A* as neighbors; Y axis shows their *AS* when with set *B* as neighbors. Functions *y=x* and *y=-x* segment figures into 4 parts. *AS* of genes with different gene sets as neighbors in smoker samples of Smoker1 data set, smoker and non-smoker samples of Smoker2 data set, smoker samples of Smoker3 data set, smoker samples of Smoker4 data set, smoker and non-smoker samples of Smoker5 data set and smoker and non-smoker samples of Smoker6 data set are shown in SFigure 1A-1I in order.

## How activity score (*AS*) is proposed?

*AS* derives from competitive gene set analysis, especially the gene set enrichment analysis (GSEA). NGP adopts the strategy of GSEA and uses enrichment score (*ES*) to score candidate genes. We modify the scoring system of NGP and propose a corrected *Z* score of the rank sum test statistic, named activity score(*AS*), to score candidate genes. *AS* is formulated as below,


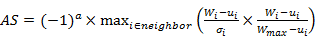
,

where $W_{i}=\sum_{j\in A_{i}} rank(z_{j})$,$u_{i}=\frac{i(N+1)}{2}$,$\sigma_{i}=\sqrt{\frac{i(N-i)(N+1)}{12}}$,
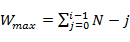
, $Z_{j}$ is the *Z* score of probe sets’ differential expression by the rank sum test, $N$is the number of probe sets on the chip, $\frac{W_{i}-u_{i}}{\sigma_{i}}$ is the *Z* score of the rank sum test statistics which indicates whether the differential expression of genes in neighbor and non-neighbor sets is different, $\frac{W_{i}-u_{i}}{W_{\max}-u_{i}}$ is a factor for adjusting the different sizes of co-regulated neighbors of candidate probe sets. If
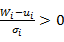
 parameter
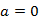
, else
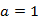
.

Activity score (*AS*) is the product of a *Z* score and a correction factor for different sizes of co-regulated neighbors. The largest possible *Z* scores of candidate genes with different sizes of neighbors in a simulation experiment are shown in Supplementary Table 1. The possible *Z* scores of candidate genes with different neighbors have different ranges, which suggest the possibility that a candidate gene with a large neighbor set including few differentially expressed genes might have higher *AS* than a candidate gene with a small neighbor set including many differentially expressed genes. We introduce a correction factor which relates to the ratio of the true *Z* score of a candidate gene with the max possible *Z* of the candidate genes ($\frac{Z}{Z_{\max}}$) to correct the bias caused by the sizes of neighbors. We demonstrate the effect of the correlation by simulated experiments. The assumed candidate genes are described in Supplementary Table 2. One hundred examples of each type of candidate genes in Supplementary Table 2 are created. We calculate *AS* of the candidate genes without correction ($(\frac{Z}{Z_{\max}})^{0}$) or with different types of corrections ($(\frac{Z}{Z_{\max}})^{1}$, $(\frac{Z}{Z_{\max}})^{2}$, $(\frac{Z}{Z_{\max}})^{3}$ and $(\frac{Z}{Z_{\max}})^{4}$). The corrections have no impact on *AS* and DE neighbors of a candidate gene when neighbors of the candidate gene are differentially expressed (Supplementary Figure 2A and 2B). However when the neighbors mix differentially expressed genes and non-differentially expressed genes, the corrections have large impacts on *AS* and DE neighbors of the candidate genes (Supplementary Figure 2C-2L). The stricter the correction is, the lower *AS* is. The candidate gene with a small neighbor set including many differentially expressed genes tends to have higher *AS* than the candidate genes with large neighbor sets including few differentially expressed genes under the corrections (Supplementary Table 3). In this paper, we correct *AS* with the correction factor $(\frac{Z}{Z_{\max}})^{1}$.

Although it has been corrected, the possible *AS* of candidate genes with different neighbor sizes still have different ranges. We study the distribution of *AS* of candidate genes with different neighbor sizes on the condition that the neighbors are randomly selected. We investigate *AS* of 100 candidate genes whose neighbors vary from 1 to 100 genes in a simulated experiment. The number of genes in the experiment is 10,000. We randomly select neighbors of the 100 candidate genes and calculate their *AS*. We repeat the process 1,000 times. Mean and standard deviation of absolute *AS* of candidate genes with different neighbor sizes are shown in Supplementary Figure 3. We calculate mean and standard deviation of absolute *AS* but not *AS* itself because *AS* is symmetric at 0. The mean and standard deviation of random absolute *AS* are quickly constrained with the neighbor sizes getting larger. Thus we directly compare *AS* of candidate genes with different neighbors because they have similar *AS* background distribution when their neighbors are large.

**STable 1 Max possible *Z* scores of** candidate genes with different neighbor sizes

| Candidate gene index | Neighbor size | Ranks of neighbors’ differential expression | *Z* |
| --- | --- | --- | --- |
| 1 | 1 | 1 | -1.7319 |
| 2 | 10 | 1-10 | -5.4756 |
| 3 | 50 | 1-50 | -12.2310 |
| 4 | 100 | 1-100 | -17.2744 |
| 5 | 200 | 1-200 | -24.3649 |
| 6 | 500 | 1-500 | -38.2153 |
| 7 | 1,000 | 1-1,000 | -53.3085 |

The number of genes in the experiments is 18981.

**STable 2 Information about the assumed candidate genes**

| Candidate gene index | Neighbor size | Ranks of differential expression of neighbors* |
| --- | --- | --- |
| 1 | 20 | 1-20 |
| 2 | 30 | 1-20 |
| 3 | 200 | 1-100 |
| 4 | 300 | 1-100 |
| 5 | 400 | 1-100 |
| 6 | 500 | 1-100 |

The number of genes in the experiments is 10,000.

The neighbors of the candidate genes (except candidate gene 1) consist of a mixture of differentially expressed genes and non-differentially expressed genes.*shows ranks of the differentially expressed genes. The non-differentially expressed genes are generated by randomly sampling from the other genes in the experiment.


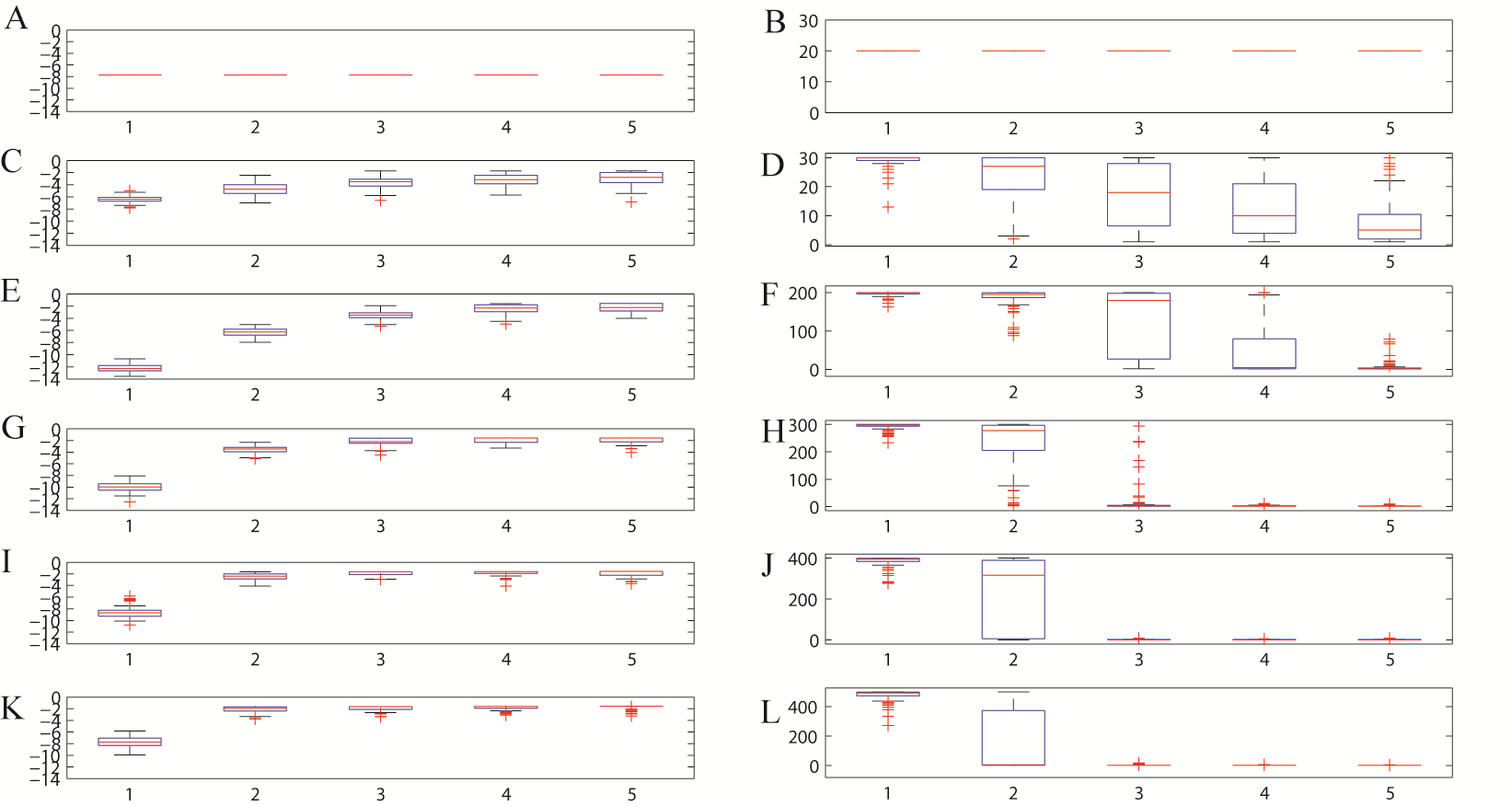


**SFigure 2 Boxplot of *AS* and DE neighbors of candidate genes under different corrections**

X axis indicates the corrections: 1-$(\frac{Z}{Z_{\max}})^{0}$, 2-$(\frac{Z}{Z_{\max}})^{1}$, 3-$(\frac{Z}{Z_{\max}})^{2}$,4-$(\frac{Z}{Z_{\max}})^{3}$ and 5-$(\frac{Z}{Z_{\max}})^{4}$. Y axis in SFigures 2A, 2C, 2E, 2G, 2I and 2K shows the value of *AS* of candidate genes. Y axis in SFigures 2B, 2D, 2F, 2H, 2J and 2L shows the number of DE neighbors of candidate genes. The *AS* and DE neighbors of candidate genes 1-6 under different corrections are shown in Figure 2A-2L in order.

**STable 3 *AS* of candidate gene 2 versus *AS* of candidate gene 3-6 under different corrections**

| Candidate gene index | $(\frac{Z}{Z_{\max}})^{0}$* | $(\frac{Z}{Z_{\max}})^{1}$ | $(\frac{Z}{Z_{\max}})^{2}$ | $(\frac{Z}{Z_{\max}})^{3}$ | $(\frac{Z}{Z_{\max}})^{4}$ |
| --- | --- | --- | --- | --- | --- |
| 3 | 1# | 1 | 0.049275405 | 7.51E-08 | 7.14E-08 |
| 4 | 1 | 1.33E-20 | 3.78E-31 | 3.18E-21 | 4.19E-16 |
| 5 | 1 | 4.43E-49 | 3.55E-47 | 3.97E-27 | 5.81E-17 |
| 6 | 1 | 1.05E-64 | 1.33E-44 | 4.92E-29 | 1.41E-21 |

The candidate genes in this table are the genes in STable 2.

In this table, we aim at comparing *AS* of candidate gene 2 with *AS* of candidate gene 3-6 under different correcting factors. *indicates that *AS* is corrected. #indicates *p* value of the t test that the mean of *AS* of candidate gene 2 is smaller than the mean of *AS* of candidate gene 3. As *AS* of candidate gene 2-6 is negative, the significant *p* values suggest candidate gene 2 has larger negative *AS* comparing to candidate gene 3-6.
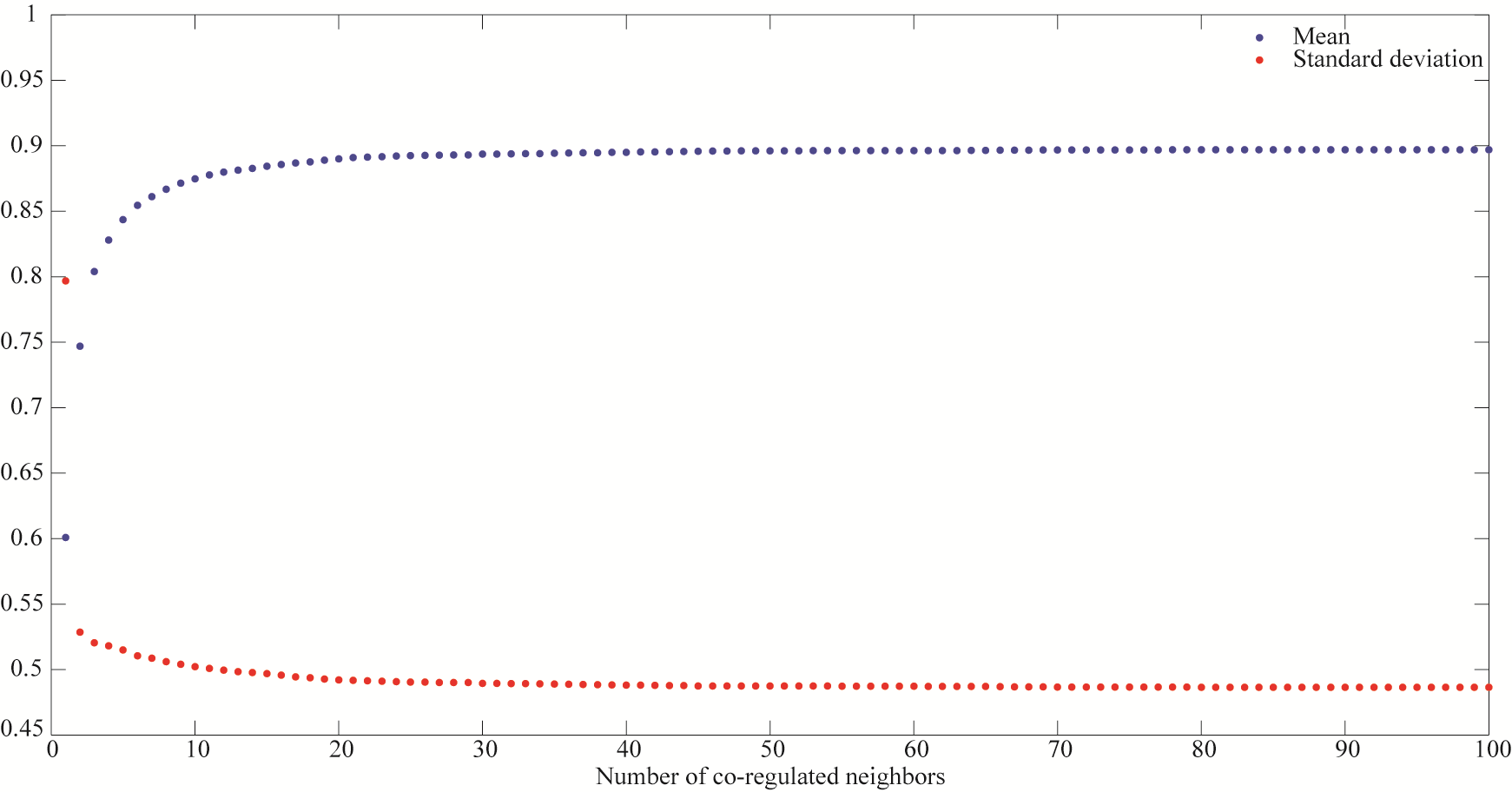


**SFigure 3 Mean and standard deviation of absolute *AS* of candidate genes with 1 to 100 neighbors**

# Advantage of nDGE comparing to NGP

How to define gene-gene relationships with regard to the phenotype under study is an important question in gene prioritization methods that integrate gene expression changes and gene-gene relationships. Two models are used in NGP to define gene-gene relationships. One is the ND model which detects gene-gene relationships that are common across different subtypes. The other is the NR model which discovers gene-gene relationships that are subtype-specific. When applying the models to prioritize genes, NGP methods are called as NGP-NR and NGP-ND respectively. NGP models effectively detect gene-gene relationships associated with phenotypes and outperform the exiting methods . However, NGP can’t accurately prioritize candidate genes with a mixture of subtype common and subtype specific neighbors.

We simulate a candidate gene with neighbors of a mixture of the ND model and the NR model in aforementioned simulated data 1. We prioritize the gene by nDGE, NGP-ND and NGP-NR and mine their DE neighbors and DE subnet. DE neighbors and DE subnet identify gene-gene relationships associated with disease in nDGE and NGP respectively. The ratios of the detected neighbors by the methods with the true neighbors are calculated. It is shown that nDGE identifies the both types of neighbors while NGP-NR and NGP-ND discover part of them (Supplementary Figure 4). nDGE prioritizes genes leveraging both types of gene-gene relationships while NGP can effectively identify only one of the two types. nDGE can produce more accurate prioritization results than NGP for the test genes.


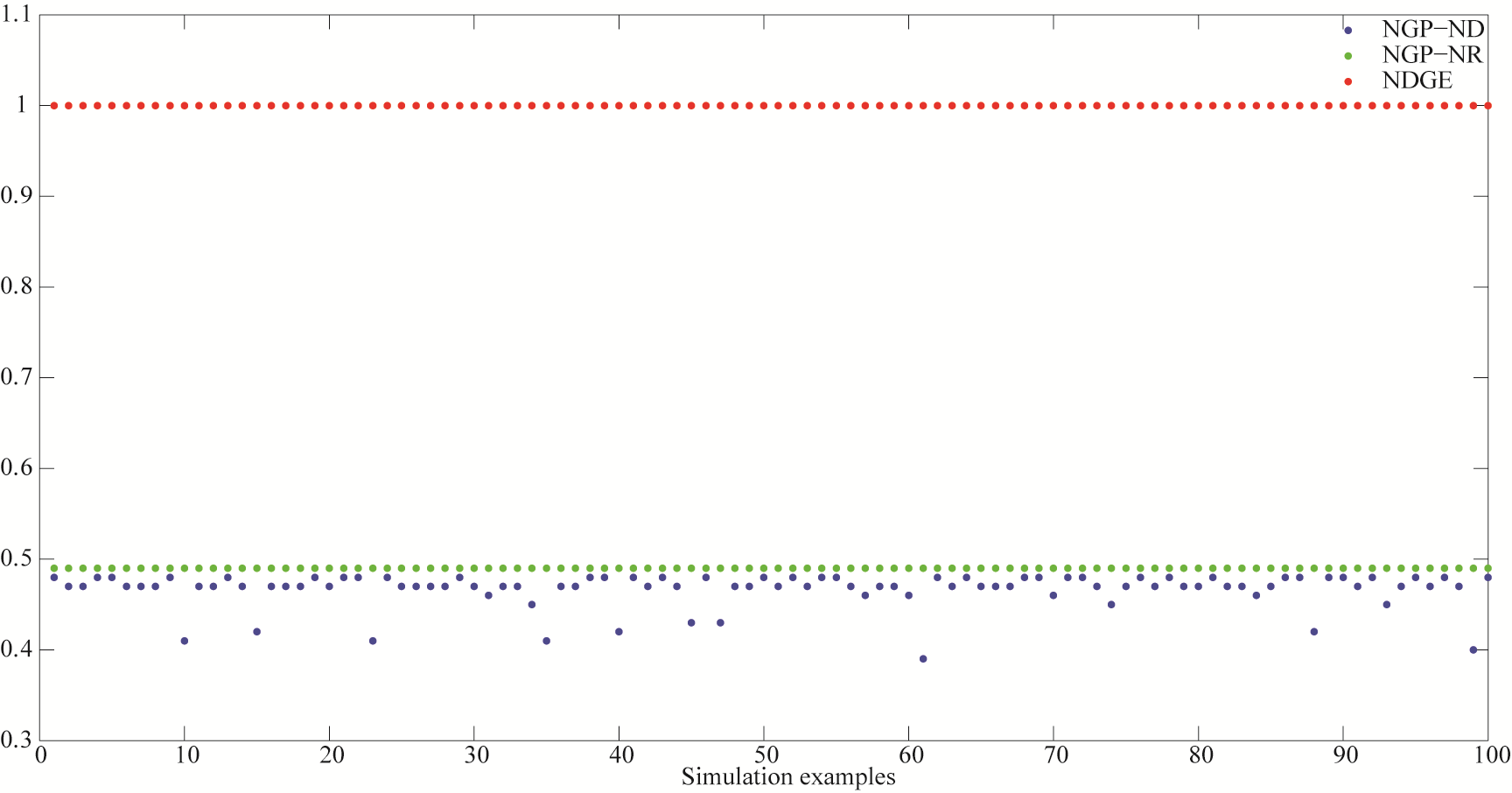


**SFigure 4 Percentage of the true neighbors detected by nDGE and NGP in 100 simulation experiments**

The scoring system for defining gene-gene relationships and gene expression changes associated with disease is the core of gene prioritization methods. NGP adapts the strategy of GSEA and uses *ES* to score candidate genes. We don’t use *ES* in nDGE because gene-gene relationships are inferred from co-expression relationships in nDGE and *ES* scores are sensitive to the selection of parameter of co-expression measurement.

We compare *AS* with *ES* in term of robustness when the parameter of co-expression measurement varies. A candidate gene is created in aforementioned simulated data set 2. nDGE produces robust scores for the candidate gene when the co-regulated neighbors are 50, 100, 150, 200, 250, 300, 350, 400, 450 and 500 (Supplementary Figure 5). The prioritization results of *AS* are more robust than that of *ES* in a large range of the parameter for neighbor size. Thus we adopt *AS* to score genes in nDGE.


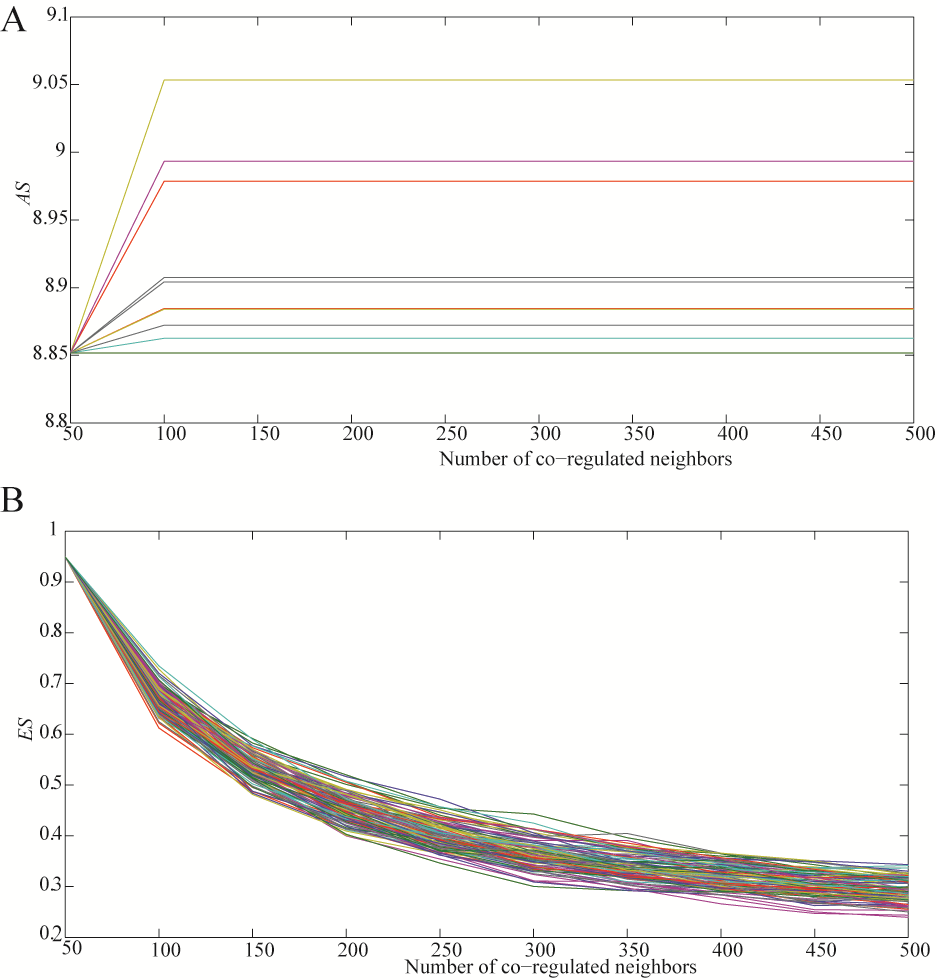


**SFigure 5 *AS* and *ES* of a candidate gene when the parameter of co-expression measurement varies in 100 simulation experiments**

A) *AS* of the candidate gene is close to constant with regard to the variation of the size of co-regulated neighbors. B) *ES* of the candidate gene varies with the variation of the neighbor size.

1. Goeman JJ, Buhlmann P: **Analyzing gene expression data in terms of gene sets: methodological issues**. *Bioinformatics* 2007, **23**(8):980-987.

2. Subramanian A, Tamayo P, Mootha VK, Mukherjee S, Ebert BL, Gillette MA, Paulovich A, Pomeroy SL, Golub TR, Lander ES *et al*: **Gene set enrichment analysis: a knowledge-based approach for interpreting genome-wide expression profiles**. *Proc Natl Acad Sci U S A* 2005, **102**(43):15545-15550.

3. Wu C, Zhu J, Zhang X: **Integrating gene expression and protein-protein interaction network to prioritize cancer-associated genes**. *BMC Bioinformatics* 2012, **13**:182.

4. Nitsch D, Tranchevent LC, Goncalves JP, Vogt JK, Madeira SC, Moreau Y: **PINTA: a web server for network-based gene prioritization from expression data**. *Nucleic Acids Res* 2011, **39**(Web Server issue):W334-338.

5. Taylor IW, Linding R, Warde-Farley D, Liu Y, Pesquita C, Faria D, Bull S, Pawson T, Morris Q, Wrana JL: **Dynamic modularity in protein interaction networks predicts breast cancer outcome**. *Nat Biotechnol* 2009, **27**(2):199-204.

6. Reverter A, Hudson NJ, Nagaraj SH, Perez-Enciso M, Dalrymple BP: **Regulatory impact factors: unraveling the transcriptional regulation of complex traits from expression data**. *Bioinformatics* 2010, **26**(7):896-904.
